# Evaluation of a Performance-Based Expert Elicitation: WHO Global Attribution of Foodborne Diseases

**DOI:** 10.1371/journal.pone.0149817

**Published:** 2016-03-01

**Authors:** W. P. Aspinall, R. M. Cooke, A. H. Havelaar, S. Hoffmann, T. Hald

**Affiliations:** 1 Aspinall & Associates, Tisbury, England; 2 Bristol University, Bristol, England; 3 Resources for the Future, Washington D. C., United States of America; 4 Delft University of Technology, Delft, The Netherlands; 5 National Institute for Public Health and the Environment, Bilthoven, The Netherlands; 6 University of Florida, Gainesville, Florida, United States of America; 7 Utrecht University, Utrecht, The Netherlands; 8 U. S. Dept. of Agriculture, Economic Research Service, Washington D. C., United States of America; 9 Technical University of Denmark, Lyngby, Denmark; US Army Engineer Research and Development Center, UNITED STATES

## Abstract

For many societally important science-based decisions, data are inadequate, unreliable or non-existent, and expert advice is sought. In such cases, procedures for eliciting structured expert judgments (SEJ) are increasingly used. This raises questions regarding validity and reproducibility. This paper presents new findings from a large-scale international SEJ study intended to estimate the global burden of foodborne disease on behalf of WHO. The study involved 72 experts distributed over 134 expert panels, with panels comprising thirteen experts on average. Elicitations were conducted in five languages. Performance-based weighted solutions for target questions of interest were formed for each panel. These weights were based on individual expert’s statistical accuracy and informativeness, determined using between ten and fifteen calibration variables from the experts' field with known values. Equal weights combinations were also calculated. The main conclusions on expert performance are: (1) SEJ does provide a science-based method for attribution of the global burden of foodborne diseases; (2) equal weighting of experts per panel increased statistical accuracy to acceptable levels, but at the cost of informativeness; (3) performance-based weighting increased informativeness, while retaining accuracy; (4) due to study constraints individual experts’ accuracies were generally lower than in other SEJ studies, and (5) there was a negative correlation between experts' informativeness and statistical accuracy which attenuated as accuracy improved, revealing that the least accurate experts drive the negative correlation. It is shown, however, that performance-based weighting has the ability to yield statistically accurate and informative combinations of experts' judgments, thereby offsetting this contrary influence. The present findings suggest that application of SEJ on a large scale is feasible, and motivate the development of enhanced training and tools for remote elicitation of multiple, internationally-dispersed panels.

## 1. Introduction

In 2007, the World Health Organization (WHO) formed the Foodborne Disease Burden Epidemiology Reference Group (FERG) with the objective of estimating the global burden of diseases acquired from food [1]. The Source Attribution Task Force within FERG aimed at determining, for each specific group of hazards, the proportion of the disease burden attributable to food and to identify—quantitatively if possible—the food commodities leading to illness [2,3]. As uncertainties may be large, defensible estimates of uncertainty in attribution were required.

**Fig 1:**
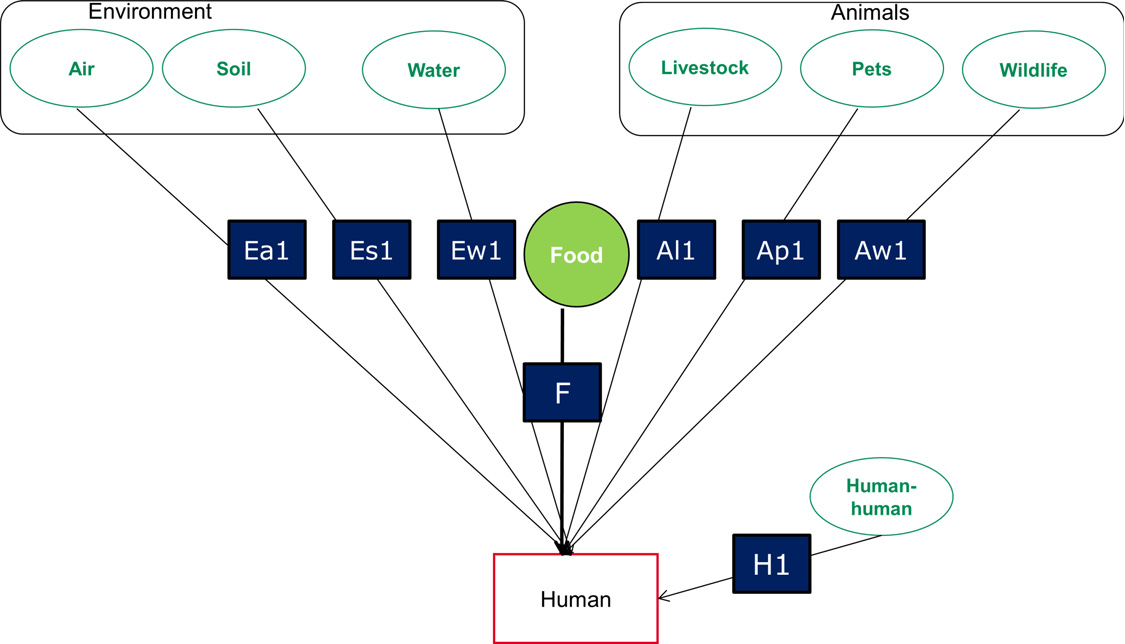
Attribution diagram. Attribution diagram: transmission routes to the point-of-exposure included in the expert elicitation [from ref. 2]. It is assumed that the pathways are mutually exclusive and exhaustive.

**Fig 2:**
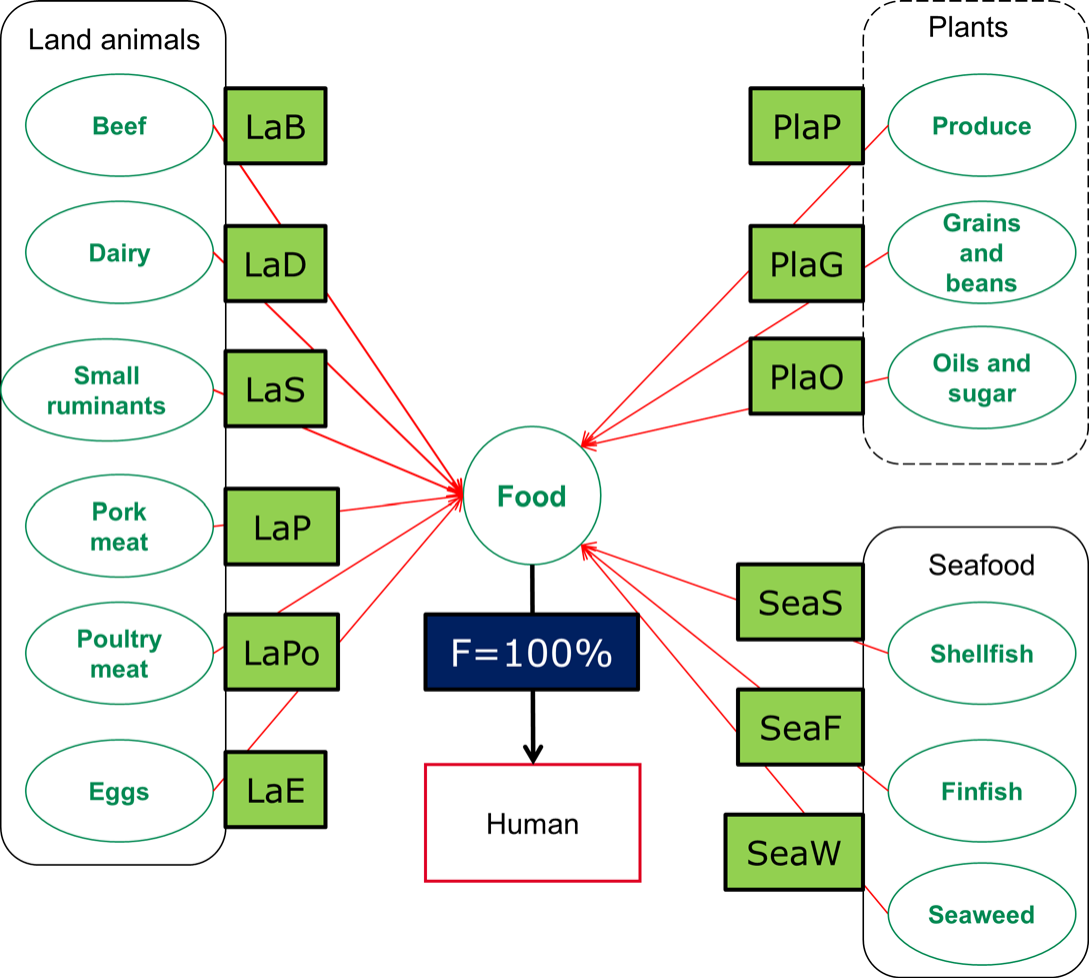
Food sub-pathways. Food sub-pathways to the point-of-entry into household, included in the expert elicitation. It is assumed that the food subpathways are mutually exclusive and exhaustive.

**Fig 3:**
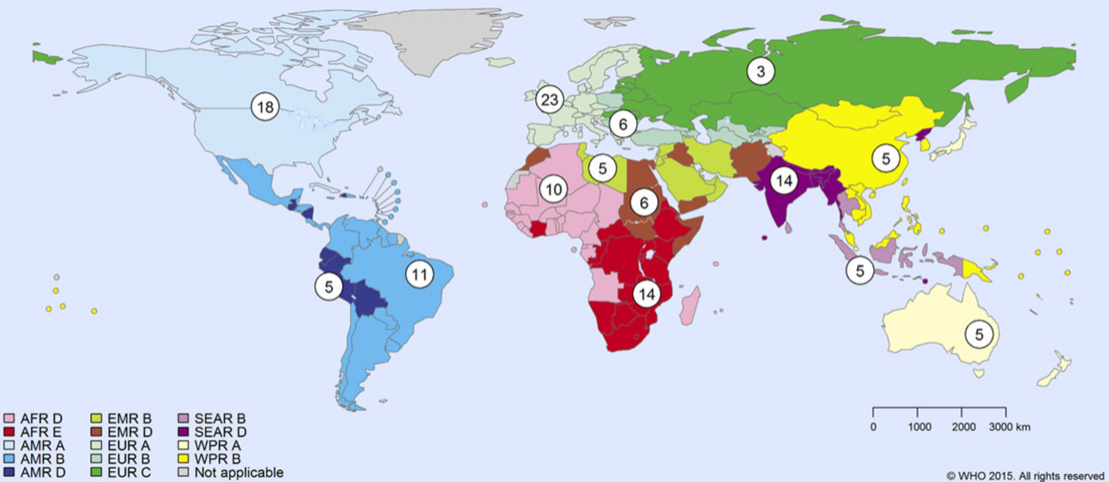
WHO sub-regions. WHO sub-regions: expert panels provided estimates for each sub-region, from [2].

The pathways by which foodborne disease hazards may reach humans are shown in Fig 1; sub-pathways are shown on Fig 2. The WHO sub-regions are shown in Fig 3.

Detailed knowledge of the pathways by which hazards reach humans affords the best opportunity for targeted intervention. However, the relative frequency with which a specific foodborne hazard exploits a given pathway will depend strongly on geography, local diet, sanitary conditions and general public health, among many other factors. Various source attribution methods exist, including microbiological and epidemiological methods, expert elicitation and methods that aim to integrate data from these approaches. Each approach has its own strengths and weaknesses [4]. Empirical data at this disaggregated level—required for informed policy interventions—is extremely sparse. Nevertheless, there are experts who understand these situations and their controlling conditions. Such knowledge is rarely, if ever, exact; it is characterized by differing viewpoints, backgrounds and experiences, and by varying levels of uncertainty. These confounding aspects of the problem, together with a lack of empirical control, have thus far discouraged the use of expert elicitation. Against this backdrop, WHO organized a structured expert judgment (SEJ) elicitation in 2013–2014, with the goal of accessing judgments from panels of experts about foodborne disease attribution in all WHO sub-regions of the world [2,3,5].

This major exercise in international expert elicitation has provided a unique opportunity to gain new insights into the challenges of using SEJ for this purpose, and to assess the strengths and limitations of the Classical Model [6] approach for pooling judgments under such circumstances. The research questions addressed in this article are:

1. *Can expert judgment provide rigorous science-based assessments of the global burden of foodborne diseases*, *in a way that is amenable to empirical control*?
2. *Are the results of the 2013–2014 WHO expert judgment assessments empirically validated in a manner comparable to other SEJ studies*?
3. *Can similar methods be deployed with scientific assurance in other data-poor circumstances*?

Companion articles [1–3,5] describe the study design, attribution findings and the results of disease burden estimates; here the focus is on the expert elicitation methodology.

## 2. Materials and Methods

Structured Expert Judgment (SEJ) is designed to convert diffuse and possibly conflicting sources of information into actionable signals of potential use to public health professionals, and to do so in a manner consistent with the best applicable scientific principles. In the WHO FERG SEJ elicitation, the Classical Model [6–9] was applied. The Classical Model is a method for deriving experts' weights based on their performance on calibration variables taken from their field, with these weights then used to aggregate judgments on target questions. To express their uncertainty judgments, experts provide three marker quantiles for each calibration and target item, and are scored with regard to statistical accuracy (measured as the p-value at which one would falsely reject the hypotheses that the probability assessments were statistically accurate), and informativeness (measured as Shannon relative information with respect to a user supplied background measure)[6].

Shannon relative information is used because it is non-negative, scale invariant, tail insensitive, slow and familiar. Slowness implies that large variations in an expert's quantile values produce only modest changes in his/her informativeness score. A difference of a factor 2 in informativeness is a very noticeable difference. Parenthetically, information measures with physical dimensions, such as the standard deviation, or the width of prediction intervals [9] raise more serious problems, as a change of units (e.g. meters to kilometers) would affect some variables but not others. Informativeness scores are not absolute, but relative to a set of experts assessing the same variables. A unique feature of the present study is that a large number of experts assessed very similar variables, allowing their informativeness scores to be compared.

Statistical accuracy is scored between 0 and 1 (higher is better) and is a very fast function. This means that the normalized product of statistical accuracy and informativeness is dominated by the statistical accuracy score. This is by design, as high informativeness should not counterbalance poor statistical accuracy. For the expert panels in the WHO data, the number of calibration variables is sufficient to distinguish good and poor statistical accuracy.

The product of statistical accuracy and informativeness constitutes the ‘combined score’ which is proportional to that expert’s performance-based weight, subject to optimization, whereby an optimal statistical accuracy threshold is chosen beneath which experts are un-weighted. The optimized weight satisfies a long-run proper scoring rule constraint whereby an expert maximizes his long-run expected score by and only by stating his true opinions.

In the Classical Model, expert responses can be combined using two forms of performance-based weights: 1) ‘global weights’, which assign an overall informativeness score to each expert based on all the calibration variables and all target questions; and 2) ‘item weights’, which modulate combination weights by using item-specific individual expert information scores. Global weights solutions were determined for the WHO study and these are referred to here as the 'Performance-Weighted Decision Maker’ or PW DM (for details, see [2,7,9]). In most SEJ applications, an equally-weighted combination of experts’ distributions is also computed (the ‘EW DM’), typically for comparison with the corresponding PW DM solutions. This provides one basis for assessing the properties and benefits of the performance-weights approach. S1 File shows anonymous expert scores and scores of performance weight (global and item) and equal weight combinations.

Any combination of experts’ assessments may be applied to the calibration variables and scored with respect to statistical accuracy and informativeness. When the calibration variables used for comparison are also used to derive the weights for PW DM, this is ‘in-sample’ testing. For ‘out-of-sample’ comparison, the weights for the PW DM are derived from a subset of variables, a training set’, and PW DM performance is measured on a separate, distinct ‘test set’ of variables. The Classical Model has been extensively reviewed both with regard to in-sample and out-of-sample performance (see [9–11] for references), and the PW DM has been shown to out-perform EW DM, both in-sample and out-of-sample [12]. Out-of-sample validation requires independent panels and intensive computations and is not practicable with the WHO data (see below). This study is concerned with the question whether an in-sample comparison between PW DM and EW DM provides similar results to those found in other SEJ studies.

### 2.1 SEJ Implementation in the WHO FERG Study

The WHO elicitation is a large-scale application of SEJ involving 72 experts from all parts of the world, distributed over 134 subject-matter panels. The sheer scale of this activity imposed many new constraints on the SEJ process whilst, at the same time, affording an opportunity to gain new insights into SEJ strengths and weaknesses.

**Fig 4:**
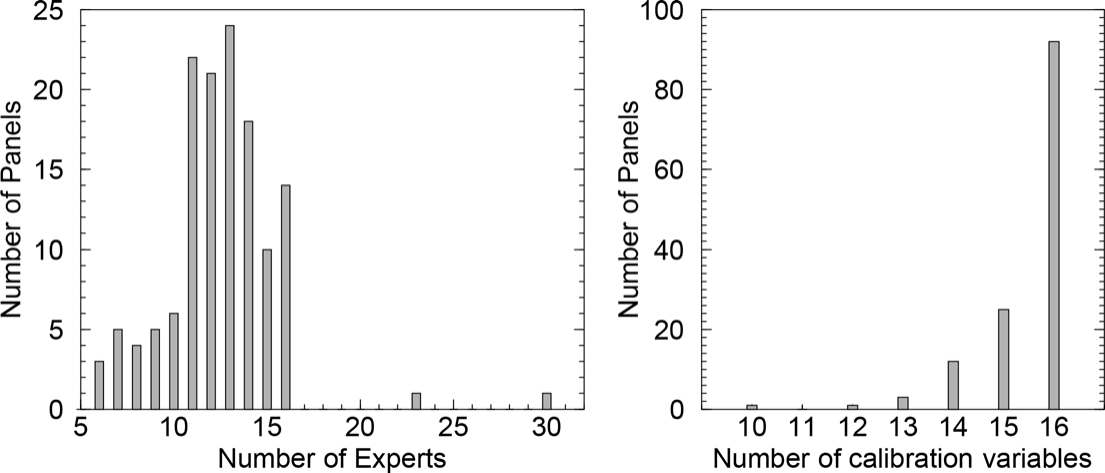
Experts and panels. Left: histogram of number of panels for number of experts; right: histogram of number of panels for number of calibration variables.

The choice of the Classical Model was largely driven by its emphasis on empirical validation with calibration variables. A secondary consideration was that the Classical Model seemed scalable to the global disease burden problem under the study constraints. Plenary meetings of the expert panels were ruled out by the geographical dispersion of the experts. Although face-to-face interviews are preferred for capturing experts' reasoning, this was also not possible given location, calendar and budget constraints. Further, to be operationally functional the elicitation protocols had to be translated into French, Russian, Spanish and Chinese. Elicitors fluent in these languages had to be identified and trained (training was conducted by S. Hoffmann and W. Aspinall). The distributions of numbers of experts per panel and number of calibration variables per panel are shown in Fig 4.

Elicitations for the calibration questions were conducted online and, for the variables of interest, experts were requested to fill in and return spreadsheets in a single online session–the goal of the exercise was to capture experts' immediate cognitive uncertainty judgments on the basis of the information provided in the questions, and not as a test of their ability to research or access relevant information or data.

The calibration variables differed by panel and were aimed at two broad domains (biological and chemical hazards). Within these domains the questions differed by specific hazard. They also included questions on food supply, child mortality, water and sanitation, disease surveillance and dietary patterns. Because of the multiple factors affecting source attribution, panels included experts from different disciplines with different areas of expertise. Reference [5] provides further detail on the calibration questions and the rationale for panels with diverse expertise. Examples of calibration questions are given in Table 1.

**Table 1:** Categories of calibration variables.

**Categories of calibration questions for biological hazards**
**Food supply**
E.g. Among all WHO sub-regions, in 2010 what was the proportion of regional vegetable supply (tonnes) that was imported rather than produced domestically in the WHO sub-region with the highest such percentage?
**Under 5 years mortality rate**
E.g. Based on WHO’s estimates, think of the country in the WHO African Region that had the largest percentage point decrease from 2000 to 2010 in all-cause under-5 mortality that was due to diarrhea. What was that percentage point *decrease*?
**Improved water and sanitation**
**Disease surveillance**
E.g. What will be the rate per 100,000 population of laboratory confirmed human cases of campylobacteriosis in 2012 in all EU member states as reported in EFSA’s annual report?
**Categories of calibration questions for chemical hazards**
**Lead**
What did the UNEP Final Review of Scientific Information on Lead report in 2010 as the mean blood lead level for children in Nigeria? Please express your answer as positive micrograms per deciliter (μg/dL)
**Cadmium**
**Inorganic arsenic**
**Dietary patterns and food supply**
E.g. Based on this FAO Food Balance Sheet data, in 2009 what was the mean percentage of rice in the national food supply available for human consumption for countries in the WHO South East Asia Region**?**

For all elicitors and for all WHO experts, this was their first exposure to this type of structured expert judgment elicitation. Combining this drawback with the remote implementation of the elicitation constituted the strongest departures from the preferred procedures for SEJ (Cooke et al., 2000). Other approaches [13], in which eliciting, say, just five distributions from a handful of experts can take up one or more days of time, would not have been feasible here; methods which do not combine experts' assessments [14], or which combine without validation [15] would have been less compliant with the empirical validation requirements of WHO.

## 3. Results

In this section, we consider how the various expert panels performed in terms of Classical Model metrics for statistical accuracy and informativeness, and assess the properties of the resulting PW DMs and EW DMs, for aggregated judgments. A panel is a group of experts who answered target questions for a particular foodborne disease hazard, either for all regions or for one specified region. Target questions asked for attribution of all cases of foodborne disease caused via primary exposure pathways (e.g. water; food; air) and via specified food sub-pathways (e.g. beef; fruits; shellfish).

There was, as a consequence, considerable overlap in membership across the panels. Overall, there were 112 panels with distinct sets of experts, though many panels differed only with respect to a few expert members. In many of these, the optimal weighted combination would assign the same weights to the same set of experts, making the PW DMs identical, even though EW DMs might differ slightly. For some hazards, the same sets of panelists provided answers for multiple regions, so the same panels could be used multiple times. Many experts participated in several distinct panels. For these reasons, any statistical analysis of results that considers the panels as independent experiments is not possible.

A recent analysis of thirty-three disjoint panels in professionally contracted SEJ studies involving 321 experts in total post-2006 [10] found that the (in sample) PW DM combined score is greater than that of the EW DM in 97% of cases. With the WHO panels, PW DM outperforms EW DM in 69% of the 112 distinct cases. This appears to be the cost of the concessions needed to implement the elicitation process, noted above.

The WHO data is distinctive in that a large number of experts assessed variables that are similar, as all of them involved relative frequencies of the same pathways and sub-pathways. The statistical accuracy and informativeness scores can be compared across all experts and across all distinct panels. This affords a unique opportunity to study the interactions of these two scoring variables.

**Fig 5:**
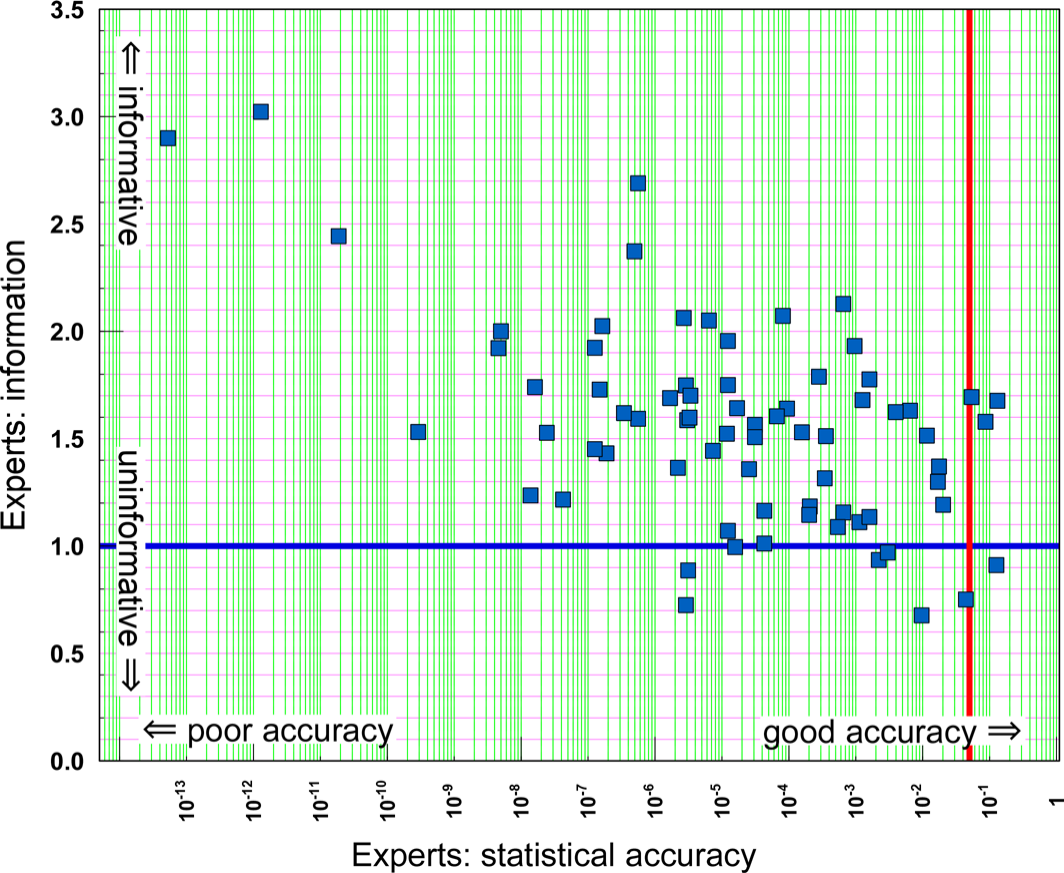
Statistical accuracy and informativeness. Statistical accuracy and informativeness for 72 WHO experts. The red vertical line demarcates the traditional 5% confidence level rejection threshold for statistical hypothesis testing, the horizontal blue line is solely for comparison with Figs 6 and 7.

Fig 5 plots the informativeness against statistical accuracy (on log scale) for all 72 WHO experts. Four of these experts achieved a statistical accuracy score above the traditional 5% confidence level rejection threshold. This is a lower fraction than is typical for Classical Model elicitations: for instance, in other SEJ cases [10], 29% of experts had a statistical accuracy score above 5%.

**Fig 6:**
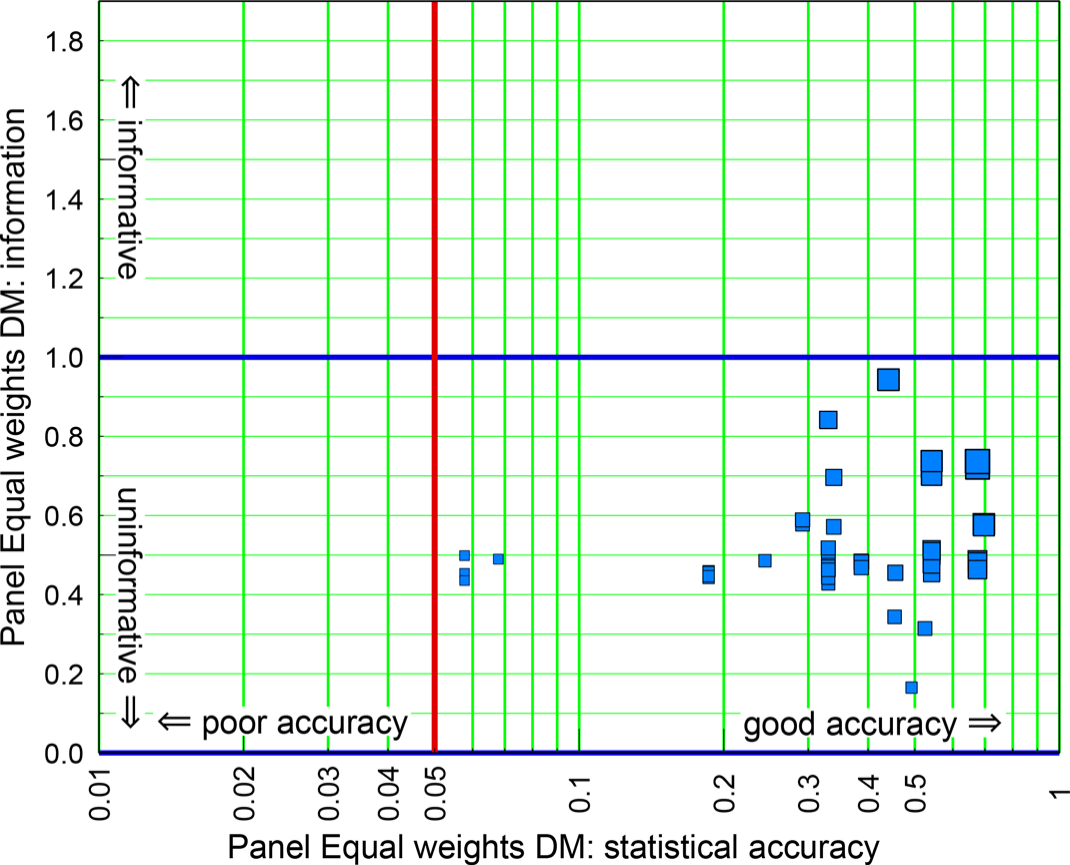
Statistical accuracy and informativeness of the Equal Weights panel EW DMs. Statistical accuracy and informativeness of the Equal Weights panel EW DMs. The vertical red line denotes the traditional 5% confidence level rejection threshold; the horizontal blue line is solely for comparison with Figs 5 and 7. Symbol size indicates Panel DM combined score: largest = 0.50; smallest = 0.035.

Fig 6 illustrates that simply averaging the distributions of all experts in each panel (the EW DM) produces distributions on the calibration variables with acceptable statistical accuracy. Although this idea has been present in SEJ folklore for some time, it is now verified here for a large set of experts assessing similar calibration variables.

Comparing the horizontal (blue) lines denoting information score equal to unity, Fig 6 also shows that statistical accuracy of the EW DM is achieved at the expense of informativeness. Whereas no single expert has an informativeness score below 0.5 this happens for 60% of the EW DMs, and none of them is above the blue line. The average EW DM informativeness score is 0.51, compared to 1.56 for the experts individually. Since informativeness is a slow function, this is a very significant reduction.

**Fig 7:**
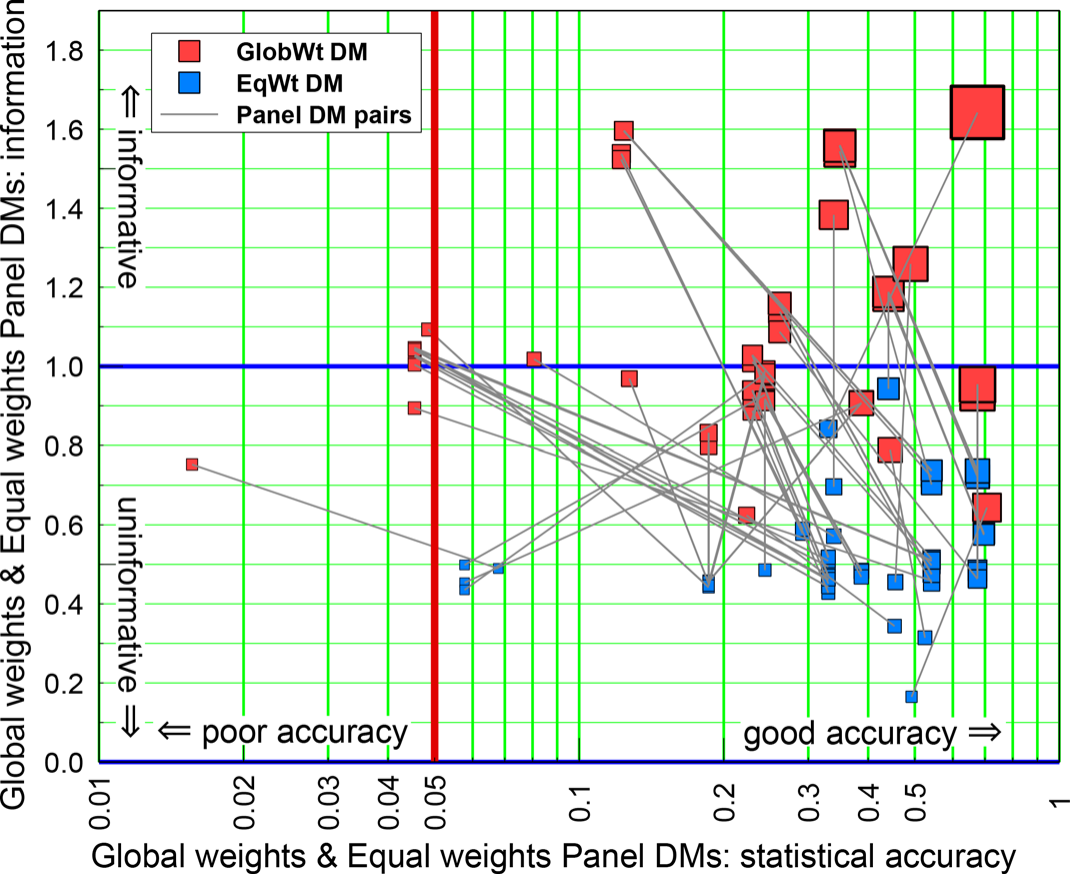
Statistical accuracy and informativeness of Performance Weights and Equal Weights Panel. Statistical accuracy and informativeness of Performance Weights (PW DM) and Equal Weights Panel (EW DM), and corresponding DM joint scores (symbol size); the thin grey lines join PW DM and EW DM solutions for individual Panels (see text). The vertical red line denotes the traditional 5% confidence level rejection threshold; the horizontal blue line is solely for comparison with Figs 4 and 5. Symbol size indicates Panel DM combined score: largest = 1.12; smallest = 0.012.

Fig 7 shows that a weighted combination of experts’ distributions, with weights based on performance, can recover much of the lost informativeness without losing much in statistical accuracy. This is indicated on Fig 7 by the lines joining panel EW DM solutions to their corresponding PW DM solutions. Some of these lines project upwards and to the right, indicating cases where the PW DM is both more accurate and more informative than the EW DM; a small majority move up to the left, indicating instances where statistical accuracy is offset to realize better informativeness.

The average informativeness score of the PW DM is 1.14, more than twice the average informativeness score of the EW DM (0.52). The statistical accuracy is degraded somewhat, relative to EW DM, but not catastrophically. On only one of the 112 panels is PW DM's statistical accuracy score below 0.045, none were below 0.01. Considering each PW DM panel as a statistical hypothesis, the statistical accuracy scores are the p-values for rejecting the hypothesis that a PW DM is statistically accurate. Rounding the p-values to two digits, only one panel would be rejected at the 5% level and none would be rejected at the 1% level. If the panels were independent (which they are not) and statistically accurate, we should expect six panels’ p-values to fall below 5%.

This large set of experts assessing multiple similar items allows a more detailed examination than hitherto of the relationship between information and statistical accuracy. The data presented in Fig 5 exhibit a negative rank correlation of -0.40 between informativeness and statistical accuracy, which is highly significant on 72 observations (the probability of this correlation, or lower, arising by chance is below 2x10^-4^), thereby confirming that statistical accuracy and informativeness are negatively correlated. Such negative correlations have been often observed in individual studies, but in those cases the relatively small numbers of experts, combined with the intrinsic non-comparability of informativeness scores, have precluded reliable quantitative conclusions.

Because of this negative association, simple weighting schemes that consider only experts' informativeness will tend to produce combinations which are very inaccurate, statistically [10]. Basing an expert’s weight only on his/her self-assessed confidence is a familiar heuristic, but one that engenders this undesirable outcome of poor statistical accuracy.

**Fig 8:**
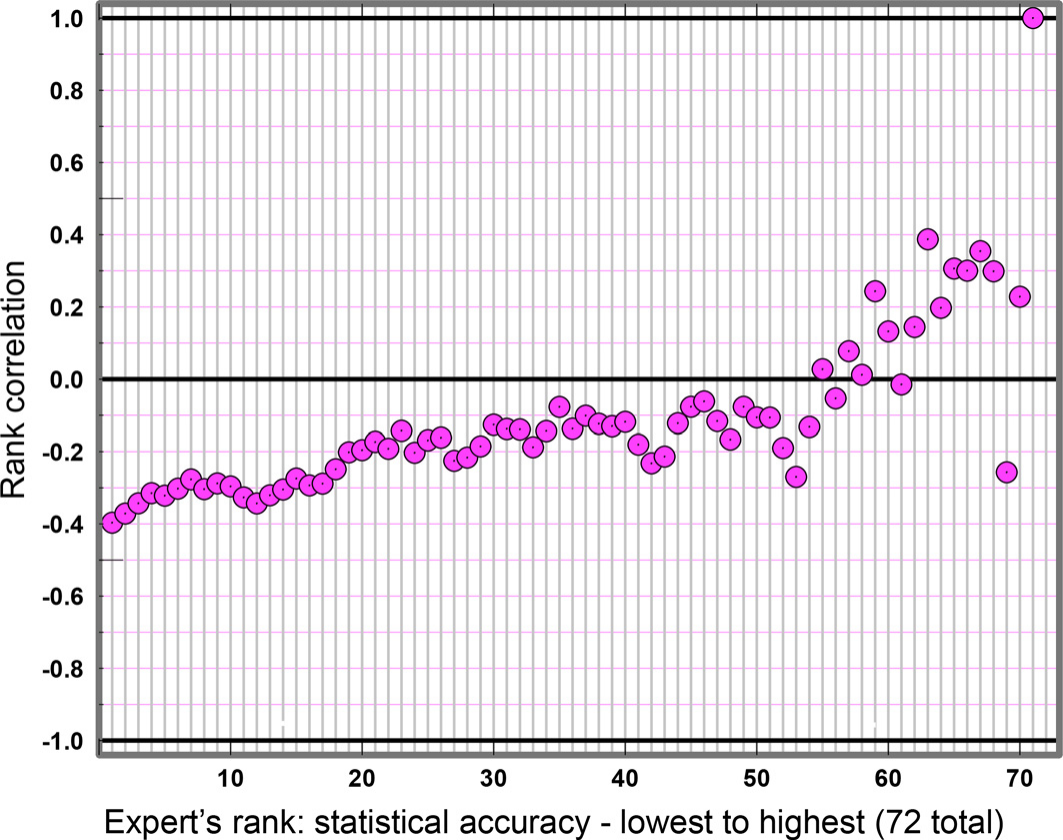
Correlation between informativeness and statistical accuracy. Running rank correlation between informativeness and statistical accuracy for Experts k = 1 … 72, with experts ordered by increasing statistical accuracy.

Another feature emerges from Fig 5, namely that the negative association between informativeness and statistical accuracy grows weaker as statistical accuracy improves. Ordering the experts from lowest to highest statistical accuracy, Fig 8 shows the rank correlation between informativeness and statistical accuracy from Expert k to 72, for k = 1 to 71. Statistical power for estimating the correlation decreases as k increases; e.g. for k = 40, there is a 10% chance of a correlation less than -0.22 arising by chance.

The negative rank correlation attenuates as the selection is restricted to experts who are more accurate statistically. In other words, the observed negative association between informativeness and statistical accuracy is driven by the *least* accurate experts.

This simple observation explains why the Classical Model, which restricts weighting to the most accurate experts, can produce PW DM combinations that are simultaneously informative and statistically accurate.

## 4. Discussion

The WHO FERG study on the global burden of foodborne diseases provides new perspectives on efficacy of SEJ. A very large number of elicitations were conducted of experts distributed around the world, by elicitors with no prior experience who had been rapidly trained. Elicitations were not conducted face-to-face. Calibration variables drawn from the experts' fields were used to gauge expert performance and to enable performance-based scoring combinations of judgments to be applied to the target items of interest. The statistical accuracies of these experts generally were lower than is typical in a dedicated Classical Model SEJ, a fact plausibly explained by the operational limitations in the present, global, elicitation process, unlike more intensive approaches.

In spite of these limitations, the statistical accuracy of both the Performance Weights and Equal Weights DM combinations were much better than that of the experts themselves, and well within the range of acceptability. Informativeness of the Equal Weights combination was strongly degraded, but informativeness of the performance-based combinations was comparable to that of the experts themselves.

Most significant in this dataset is the negative rank correlation between informativeness and statistical accuracy, and the finding that this correlation weakens when expert selection is restricted to the statistically more accurate experts.

These results motivate the development and deployment of enhanced elicitor and expert training, and advanced tools for remote elicitation of multiple, internationally-dispersed panels–demand for which is growing in many disciplines.

The demonstration that SEJ applications on this scale are feasible and potentially successful offers new options for many scientific and technical areas in which the inchoate information embedded in widely dispersed experts can be actively accessed for the benefit of decision- or policy makers.

## 5. Conclusions

The research questions addressed in this study may be answered as follows:

1 *Can expert judgment provide rigorous science-based assessments of the global burden of foodborne diseases*, *in a way that is amenable to empirical control*?

Yes. The Classical Model enables empirical control, however this control is *probabilistic*. The hypothesis being tested is not "is the assessment of an expert or combination of experts correct?", but "are the probabilistic statements of an expert or combination of experts statistically accurate?" The validation is based on calibration variables from the experts' field for which true values are known *post hoc*. Validation based on variables of interest is not possible until these variables can be observed.

2 *Are the results of the 2013–2014 WHO expert judgment assessments empirically validated in a manner comparable with other SEJ studies*?

Partially. As with other studies, equal weighting of experts per panel raised statistical accuracy to acceptable levels, but at the cost of informativeness. Performance-based weighting increased informativeness without sacrificing accuracy. For more than 95% of the expert panels, the hypothesis that these Performance Weights combinations yield statistically accurate probability statements would not be rejected at the 5% level. Whereas Equal Weights combinations were much less informative than the experts themselves, the informativeness measures of Performance Weights solutions were comparable to those of the experts. This pattern is consistent with comparable SEJ studies. On the other hand, the overall statistical accuracy of the experts in this study was lower than that found in comparable Classical Model studies.

3 *Can similar methods be deployed with scientific assurance in other data-poor circumstances*?

Yes. This study finds that the negative correlation between informativeness and statistical accuracy is attenuated as statistical accuracy improves. This augurs well for performance-based combination methods that restrict weighting to subsets with statistical accuracy, and among these, reward informativeness.

## Supporting Information

S1 File WHO expert scores. The EXCEL file contains experts’ names and scores per panel. “pwg” denotes global performance weights, pwi denotes item specific performance weights, “ew” denotes equal weights.
(XLSX)
